# Blastomycosis—Some Progress but Still Much to Learn

**DOI:** 10.3390/jof8080824

**Published:** 2022-08-07

**Authors:** Matthew F. Pullen, Jonathan D. Alpern, Nathan C. Bahr

**Affiliations:** 1 Division of Infectious Diseases and International Medicine, Department of Medicine, University of Minnesota, Minneapolis, MN 55455, USA; 2 Division of Infectious Diseases, HealthPartners, Bloomington, MN 55425, USA; 3 Department of Medicine, University of Minnesota, Minneapolis, MN 55455, USA; 4 Division of Infectious Diseases, Department of Medicine, University of Kansas Medical Center, Kansas City, KS 66160, USA

**Keywords:** *Blastomyces*, *Blastomyces dermatitidis*, blastomycosis, mycosis, diseases, fungus

## Abstract

Blastomycosis, caused by *Blastomyces* spp., is an endemic mycosis capable of causing significant disease throughout the body. Higher rates of infection are seen in the Mississippi and Ohio River valleys, the Great Lakes region of the United States and Canada, much of Africa, and, to a lesser extent, in India and the Middle East. Limited reporting inhibits our true understanding of the geographic distribution of blastomycosis. An estimated 50% of those infected remain asymptomatic. Of those who present with symptomatic disease, pulmonary involvement is most common, while the most common extrapulmonary sites are the skin, bones, genitourinary system, and central nervous system. Itraconazole is the standard therapy for mild–moderate disease. Data for other azoles are limited. Amphotericin is used for severe disease, and corticosteroids are occasionally used in severe disease, but evidence for this practice is limited. Despite increasing incidence and geographic reach in recent years, there are still significant knowledge gaps in our understanding of blastomycosis. Here, we provide an updated review of the epidemiology, clinical presentations, and diagnostic and therapeutic approaches for this infection. We also discuss areas needing further research.

## 1. Introduction

Endemic mycoses are fungal pathogens known to cause human disease and that have characteristic geographic distributions [1]. These fungi are increasingly important in aging and vulnerable populations, such as post-transplant or otherwise immunocompromised persons [2,3]. Among the endemic fungi, *Blastomyces* is relatively rare, but it has potentially devastating consequences. Mortality data are fairly limited due to gaps in disease reporting for blastomycosis, yet, case fatality rates of 4–22% have been reported, depending on patient demographics, such as advanced age, male sex, ethnicity, and living in hyperendemic regions [4]. Thus, knowledge of blastomycosis is incredibly important for clinicians in hyperendemic areas and those seeing patients who have traveled to such areas. In addition, we must continue to be aware that areas of endemicity are shifting for blastomycosis and other endemic mycoses [5]. The primary etiologic agents of blastomycosis are *Blastomyces dermatitidis* species complex (comprised of *B. dermatitidis* and the more recently described *Blastomyces gilchristii*) and *Blastomyces helicus.* In the Middle East and Africa, *Blastomyces percusus* and *Blastomyces emzantsi* are uncommon causes of blastomycosis; a newer member of the *Blastomyces* genus, *Blastomyces parvus*, has also been described as a rare cause of atypical, granulomatous pulmonary blastomycosis [6,7,8].

While most commonly seen as a pulmonary pathogen, *Blastomyces* spp. can disseminate throughout the body, often mimicking other infections and/or malignant processes [9,10]. Given the syndromic similarity of *Blastomyces* infection to other disease processes, a high index of clinical suspicion is crucial to ensure the prompt recognition and treatment of blastomycosis. There are disproportionately little recent data on blastomycosis, particularly regarding the increasing geographic spread of these fungi and the utilization of new antifungal agents [2,5,11]. In this review, we examine the current data on the epidemiology, clinical presentation, and treatment of blastomycosis and comment on particularly important areas where additional research is urgently needed.

## 2. Epidemiology

The natural environment of *Blastomyces* is moist, acidic soil, particularly in vegetation-dense areas near rivers or other water sources. Early efforts to isolate *Blastomyces* in soil samples were largely based on culture or recovery from intravenous injection of soil samples into mice, often a difficult process with few successes. With the advent of PCR technology, nucleic acid detection has successfully identified *Blastomyces* in the expected ecologic niche, that is, moist soil with dense woodland or vegetation nearby, or from tissues of animals exposed to these environments [12,13,14,15,16,17,18]. Thus, *Blastomyces* is often reported in persons who work outdoors, hunt or fish, or have recent exposures to areas where soil and vegetation have been disturbed (construction, excavation, etc.) [5,19]. Reinforcing this soil–spore connection, the risk of blastomycosis has been shown to be higher in canines, particularly sporting or hunting dogs, exposed to these endemic regions, which is likely due to their closer proximity to the soil and digging/sniffing behaviors [20,21]. *Blastomyces*, being a thermally dimorphic fungus, grows as a filamentous, mycelial mold form in the natural environment, undergoing a phase transition into a pathogenic budding yeast form in warmer environments, such as the human (or other mammals) body, capable of evading host immune defenses to cause both pulmonary and disseminated infection [22].

**Figure 1:**
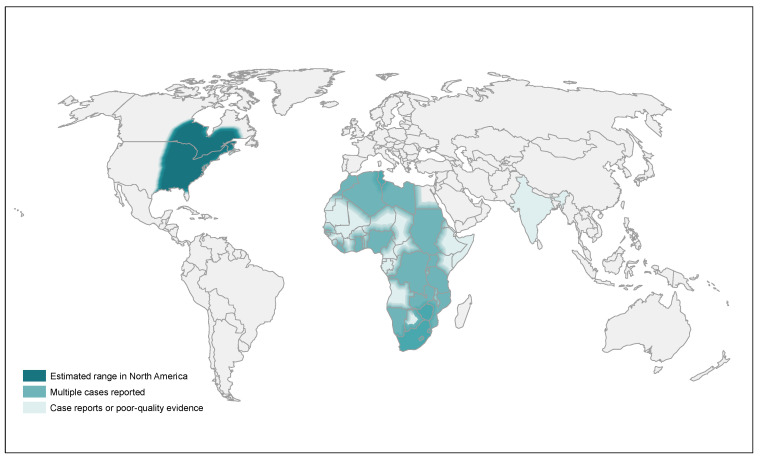
Map of the estimated distribution of *Blastomyces*. Figure used with permission from Ashraf et al. [5].

Within the United States, *Blastomyces* is typically considered endemic within the states along the Ohio and Mississippi River Valleys and Great Lakes region, with extension into several central and eastern provinces of Canada (Ontario, Quebec, Manitoba, and Saskatchewan), which has been observed in both earlier and more recent epidemiologic studies (Figure 1) [5,23,24,25,26]. Presently, blastomycosis is a reportable infection in only five states within the United States (Arkansas, Louisiana, Michigan, Minnesota, and Wisconsin) [27]. While these states do represent regions with a historically higher incidence of blastomycosis, the relative lack of data from neighboring states raises questions about the true incidence outside of known endemic regions, particularly in light of case reports of blastomycosis in non-endemic areas dating back decades [26,28,29,30]. The bulk of epidemiologic data predate the identification of *B. gilchristii*, which is included in the *B. dermatitidis* complex designation, though some studies suggest that *B. gilchristii* is more geographically restricted to Canada and the northern United States region [31]. By comparison, *B. helicus* has been more commonly isolated in the western United States (Colorado, Idaho, Montana, California, Nebraska, Texas, and Utah) and western Canada (Alberta and Saskatchewan) [5,32].

Outside of North America, blastomycosis has been widely reported across Africa, though as is the case in much of North America, it is not a commonly reportable infection, so epidemiologic data are often limited to case reports [5,8,33,34,35,36,37,38,39,40]. Though less often reported, blastomycosis has also been seen in the Middle East and India, but differences in disease patterns and a recent molecular examination of several cases suggest that, in these regions, two separate species (*Blastomyces percusus* and *Blastomyces emzantsi*) may represent a significant portion of the disease burden [8,41,42].

The non-reportable status of blastomycosis in much of the world, as well as a dearth of broader epidemiologic studies of this infection, make it difficult to truly assess the emerging geographic spread of *Blastomyces*. Couple this with the possible expansion of endemic fungi domains due to climate change, and it is easy to see that more study is needed on the true incidence and geography of these infections [43].

## 3. Clinical Presentation

Prior studies of point-source outbreaks of blastomycosis have suggested that an estimated 50% of persons infected with *Blastomyces* after an exposure experience minimal to no symptoms [44,45]. In those who go on to develop symptomatic infection, the typical incubation period is 4–6 weeks [46]. As expected, due to the inhalational route of exposure, pulmonary infection is the most common symptomatic presentation, accounting for at least three-quarters of diagnosed blastomycosis cases [47]. Amongst the *Blastomyces* spp., those within the *B. dermatidis complex* infection are more common in immunocompetent individuals and more often cause pulmonary symptoms compared to *B. helicus* infection, which is seen more often in immunocompromised persons and as a systemic disease [32]. Those species more common outside of the United States (*B. percusus* and *B. emzantsi*) seem to have a proclivity for dissemination from the lungs to cutaneous and osseous sites, though further studies are needed to solidify this pathogen–presentation relationship [7].

### 3.1. Pulmonary Blastomycosis

The most common presentation of symptomatic pulmonary blastomycosis is an indolent, chronic process, with patients typically complaining of weight loss, intermittent low-grade fever, chest pain, fatigue/malaise, dyspnea, and cough (often with scant sputum production and occasionally associated with hemoptysis) [9,10,47,48,49]. Less commonly, patients present with (or progress to) an acute form of pulmonary infection. In its acute form, pulmonary blastomycosis presentations can range in severity from subclinical pneumonia to acute respiratory distress syndrome (ARDS) [9,47,50]. Typical symptoms include fever, night sweats, dyspnea, cough (productive or non-productive), hemoptysis, fatigue, malaise, anorexia, and weight loss, often leading to an initial misdiagnosis of bacterial pneumonia [10,48,49]. Of those who rapidly develop ARDS due to acute pulmonary blastomycosis, mortality approaches 50% and is particularly severe if there was a diagnostic delay [46,47].

**Figure 2:**
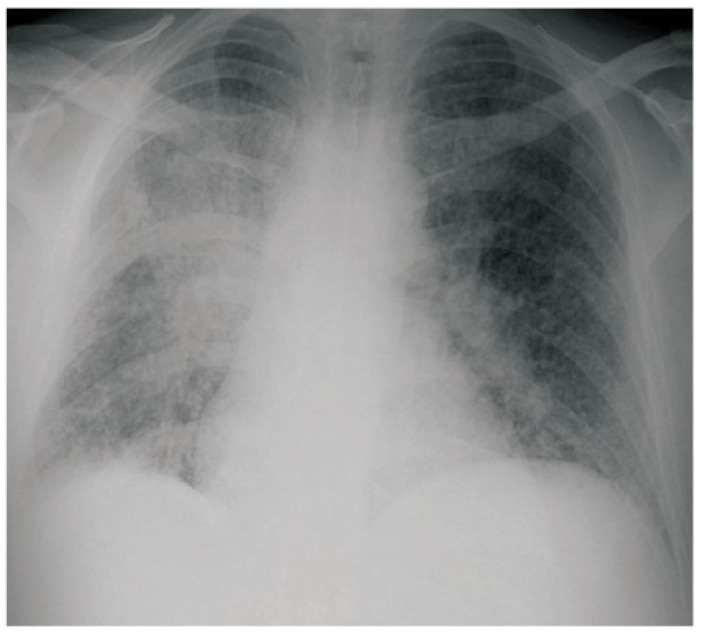
Pulmonary blastomycosis chest X-ray image. Chest X-ray seen in a patient with pulmonary blastomycosis demonstrating a right lower lobe consolidation and bilateral military nodules. Image sourced from Sarkar et al. under a creative commons license (CC BY 3.0) [56].

Imaging findings in these syndromes are variable and nonspecific. The most common presentation on chest imaging is airspace consolidation, most often described as patchy opacities. Less commonly, in fulminant pulmonary blastomycosis, large and/or bilateral consolidations can be found [10,50,51]. Other less common radiographic findings include mass-like lesions [52,53], granulomatous or cavitary lesions similar to tuberculosis [54,55], nodules [10,51], diffuse interstitial “tree-in-bud” nodularities, or, rarely, miliary disease with endobronchial extension. Many of these findings are more common when focal airspace opacity is also present. Figure 2 represents the more common, non-specific chest X-ray findings seen in pulmonary blastomycosis. The wide spectrum of radiographic findings in pulmonary blastomycosis often leads to misdiagnosis as other pulmonary infections or syndromes, such as bacterial pneumonia, sarcoidosis, tuberculosis, and malignancy [9,10]. Slow or no response to typical therapy for these conditions should raise suspicion for blastomycosis, particularly with the patient history.

**Figure 3:**
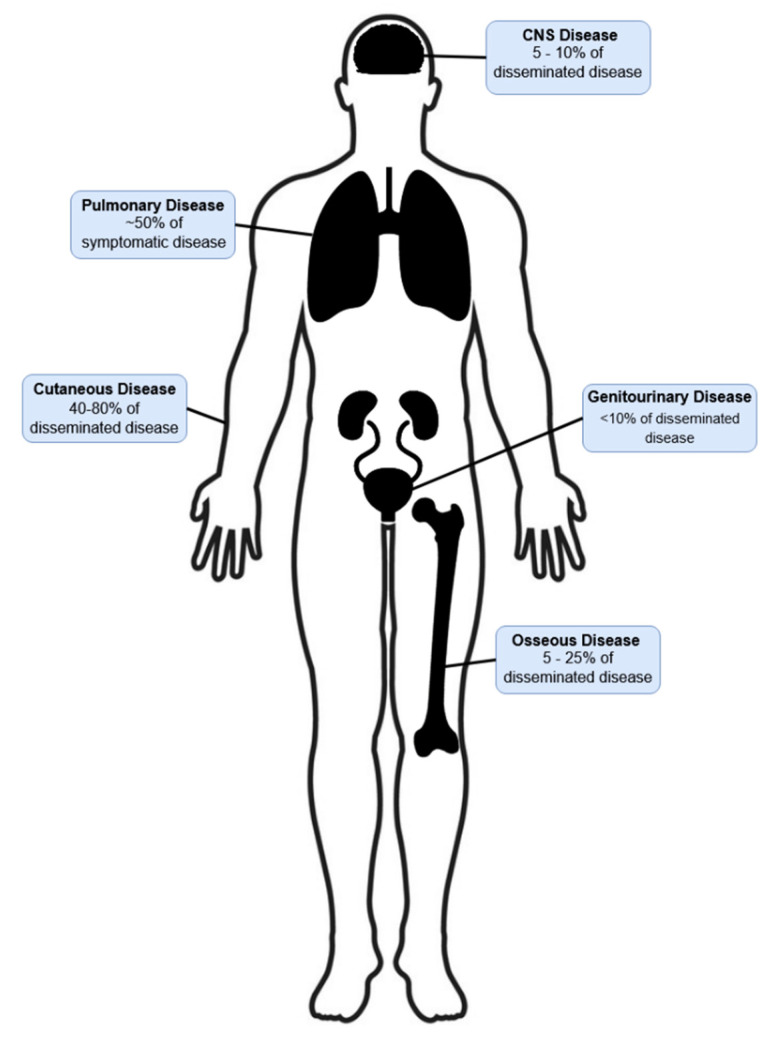
Sites of disseminated blastomycosis. CNS: central nervous system.

Of those who develop symptomatic infection, an estimated 25–40% develop extrapulmonary disease, most often disseminated from pulmonary disease (though direct inoculation can rarely occur) [46,47]. The most commonly involved extrapulmonary sites are the skin (40–80% of disseminated disease), bones (5–25%), and the genitourinary system (less than 10%) [46]. Central nervous system infection is rare, occurring in 5–10% of disseminated disease, with immunocompromised populations at higher risk [47,57]. Figure 3 summarizes the common sites of dissemination and the frequencies of dissemination to these sites.

### 3.2. Cutaneous Blastomycosis

**Figure 4:**
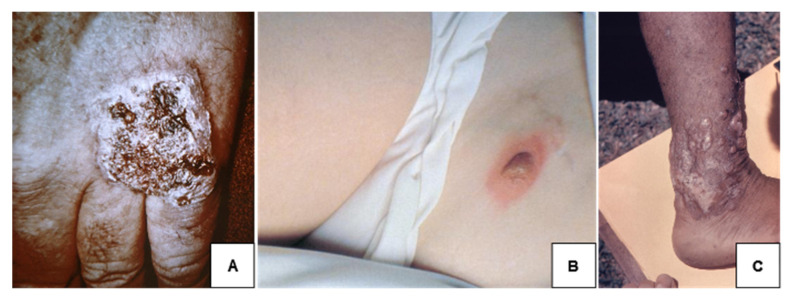
Cutaneous blastomycosis. (**A**) Verrucous blastomycosis; (**B**) nodular cutaneous blastomycosis with bulla formation; (**C**) keloidal blastomycosis. All images were obtained through the CDC Public Health Image Library (https://phil.cdc.gov (accessed on 1 August 2022)).

Skin is the most common site of extrapulmonary blastomycosis, typically presenting initially as papulopustular lesions. These commonly progress to warty, verrucous plaques with heaped margins or, less often, lesions with central ulceration, abscesses, or violaceous nodules [58,59]. Severe cutaneous blastomycosis can expand to hundreds of lesions (typically not the scalp) and may also cause osteomyelitis by direct extension [47,58,59]. Mucus membranes are less commonly affected, though laryngeal, oral, and nasal lesions have been reported [60]. Similar to the pulmonary form of blastomycosis, the cutaneous form is often misdiagnosed as malignancy (basal or squamous cell carcinoma); bacterial infection (tuberculosis); or other skin processes, such as pyoderma gangrenosum or keratoacanthoma [60,61]. Rarely, isolated cutaneous blastomycosis without concurrent pulmonary infection is thought to occur related to a resolved prior pulmonary infection or direct inoculation; these infections are usually more limited [59,62]. Figure 4 shows several different cutaneous presentations of blastomycosis.

### 3.3. Osseous Blastomycosis

Osteomyelitis due to blastomycosis is typically associated with extension into the surrounding tissues causing abscesses, sinus tracts, and septic arthritis of adjacent joints [47,63]. Prior case series have shown a predilection for lower extremities and the lumbar or thoracic spine, though virtually any bone can be involved [47,61,64,65,66]. Lesions may or may not cause pain and may mimic cancers either as bone masses or lytic lesions [63,67,68]. Concurrent pulmonary disease is common but not universal [47,61,64].

### 3.4. Genitourinary Blastomycosis

Blastomycosis can also affect the genitourinary system via dissemination (though it has been rarely described in isolation) [69,70]. In men, the two classic syndromes are prostatitis +/−abscess and epididymitis [47,71]. Typical symptoms of *Blastomyces* prostatitis include urinary obstruction, dysuria, hematuria, hematospermia, and perineal or suprapubic pain [70,72,73]. Similarly, epididymitis typically presents with pain in the affected region, along with scrotal and/or testicular swelling, with rare sinus tract formation [47]. Genitourinary disease in female patients has been described less commonly but most often presents as tubo-ovarian abscesses, endometritis, and salpingitis [47,74,75,76].

### 3.5. Central Nervous System Blastomycosis

CNS blastomycosis typically presents in the setting of multisystem disease (isolated CNS disease has also been rarely reported) [77,78,79]. Common presentations include a typical meningitis pattern (headache, nuchal rigidity, or other signs of meningism). Space-occupying lesions can occur within the cranium or spine and may cause abscesses [61,77,80,81]. True to its “great mimic” reputation, there are several case reports highlighting the frequent misdiagnosis of *Blastomyces* meningitis as tuberculous meningitis [82,83,84].

In all forms of blastomycosis, a common theme is the mimicry of other conditions [9,10,82,83,84]. Thus, particularly after treatment failure for more common diseases, blastomycosis should be strongly considered. In a patient with proper geographic- or exposure-related risk factors, blastomycosis should be considered immediately. Though pulmonary disease is most common (and is typically present in those who also have extrapulmonary disease), extrapulmonary forms are highly morbid and can also be fatal [46,47].

## 4. Diagnosis

The epidemiology outlined and history taking (for example, outdoor activities, such as chopping wood or digging into dirt, or even exposure to a construction site that disrupts soil or decaying wood) are crucial considerations that must play a part in the diagnosis of blastomycosis [5,19]. Interestingly, a patient’s dog having been diagnosed with *Blastomyces* infection may be another helpful clue [85]. Of course, when ordering diagnostic tests, the symptoms being experienced by the patient affect which test(s) might be helpful. Regardless of whether sputum or other tissues are used, histopathologic techniques are crucial to the prompt diagnosis of blastomycosis, and early use may allow for the avoidance of diagnostic delays [86].

**Figure 5:**
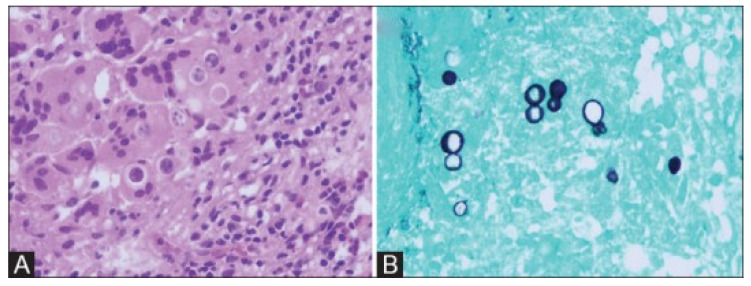
Characteristic *Blastomyces* microscopy. Hematoxylin and eosin staining of a *Blastomyces*-containing cerebellar mass at ×400 magnification (**A**) with large, round, thick-walled yeast cells. Grocott’s methenamine-silver-stained section of the same mass at ×400 magnification (**B**) with broad-based budding yeast cells. Image source is Kochar et al., 2016, used under a creative commons license (CC BY-NC-SA 3.0) [92].

*Blastomyces* yeast is typically described as broad-based budding, and it is 8–20 µM in diameter with a doubly refractile cell wall [87]. Interestingly, giant forms have been described occasionally that may be confused with *Coccidioides* [88]. Stains such as Gomori methenamine silver, calcofluor white, periodic acid–Schiff, and 10% potassium hydroxide are commonly used to visualize *Blastomyces* yeast [47,89,90]. Sputum staining with potassium hydroxide is of particular interest in that it is a rapid method (15–30 min) with a fairly high sensitivity (36–90%) [89,91]. Obviously, this method does not identify all cases, but when used up front, it may allow for prompt diagnosis and treatment. Figure 5 demonstrates the common microbiologic findings of *Blastomyces*, notably broad-based budding yeast cells. In disseminated blastomycosis, examination of skin tissue samples may be quite useful, and these techniques can also be utilized on bone, blood, cerebrospinal fluid (CSF), or prostatic tissue samples depending on the site(s) involved.

Fungal culture is another traditional method of diagnosis. While often more sensitive, culture may take up to five weeks to grow *Blastomyces* (though one or two weeks is more common) [87]. One interesting study found cultures from different respiratory fluids to have different yields, although they were limited to some degree by small numbers. Generally, respiratory cultures seem to have sensitivities >70%, with exact numbers depending on the fluid type. Culturing multiple specimen types (tracheal secretions, sputum, and bronchoalveolar lavage (BAL) fluid) seems to be additive [87,91]. A DNA probe (AccuProbe, Hologic, Inc., San Diego, CA, USA) is also available to be used on cultures growing fungi that have not yet been identified; however, this may cross-react with *Emergomyces* spp. [93].

*Blastomyces* spp. galactomannan antigen detection by an enzyme immunoassay is also commercially available and can be used on urine, serum, BAL fluid, and CSF [93]. This technology is relatively rapid (though for some assays, shipping slows turn-around time). Urine antigen testing is generally the most sensitive (75–90% depending on disease severity, the location of infection, assay type, and the laboratory performing testing), and levels may correlate with disease severity [94,95,96,97,98]. BAL fluid may have similar sensitivity (80%), although one study found a sensitivity closer to serum, wherein the EIA generally performs more poorly (50–60%) [94,95,96,99]. Similar to the potassium hydroxide staining of pulmonary fluid samples, if positive, these tests are quite helpful, but a negative test cannot rule out blastomycosis. One study found higher sensitivity in immunocompromised persons than in those without immune deficits, presumably because of higher antigen loads in such patients [100]. Importantly, broad cross-reactivity occurs with members of the *Histoplasma*, *Talaromyces*, and *Paracoccidiodes* genera [89,95,96,101].

Complement fixation and immunodiffusion antibody detection methods have been available for some time but are not frequently used given variable (and generally poor) reported performance characteristics [87,98]. However, a new EIA detecting antibodies against BAD-1 (a cell wall adhesion antigen) has shown higher sensitivity (88%) and specificity (94–99%) and, thus, may have some role. Importantly, this test is not currently available commercially despite the initial study having been published in 2014 [102].

Nucleic acid amplification tests, such as polymerase chain reaction (PCR), have been developed for *B. dermatitidis* but are not commercially available. Potential advantages could be use on tissue (possibly including paraffin-embedded tissue) or body fluids and without cross-reactions with other fungi [93,103]. Broad-range fungal PCR and metagenomic next-generation sequencing (mNGS) certainly can detect *Blastomyces*, and the use of mNGS has been reported in a patient suspected to have tuberculosis or lung cancer. Ultimately, that patient was found (via tissue mNGS) to have *B. dermatitidis* infection [104]. However, the role of these technologies for blastomycosis is not clear at this time.

## 5. Treatment

Updated Clinical Practice Guidelines for the treatment of blastomycosis were published by the Infectious Disease Society of America (IDSA) in 2008 [46]. No updates to this guideline have been published in over a decade, which is likely due to the uncommon occurrence of blastomycosis and the resultant dearth of large, well-run clinical trials. With the rare exception of asymptomatic infection or mild pulmonary blastomycosis in immunocompetent hosts that improve clinically and radiographically prior to diagnosis [105,106], all cases of blastomycosis should be treated to prevent disease progression [46]. The approach to treatment generally depends on the site of infection, disease severity, and the immune status of the patient, as summarized in Table 1. Additional factors that should be weighed include drug-related toxicities and patient co-morbidities.

### 5.1. Pulmonary Blastomycosis

The choice of initial therapy for pulmonary blastomycosis depends on the severity of disease. Unfortunately, there are limited data available to guide clinicians in the assessment of disease severity, which is often left to clinical judgement [46]. This remains an important area of further study.

#### 5.1.1. Mild-to-Moderate Pulmonary Blastomycosis

Mild-to-moderate disease is usually treated with itraconazole 200 mg three times per day for three days, followed by 200 mg once or twice daily for 6–12 months. Treatment is often continued for a few months after clinical and radiographic resolution, though more data are needed to support this practice and to determine the optimal duration of therapy [46]. The disadvantages of itraconazole include extensive drug–drug interactions, drug-related side effects, and absorption issues. The oral suspension formulation of itraconazole should be taken on an empty stomach and is preferred due to a more predictable absorption and improved bioavailability compared to the capsule formulation [107]. However, gastrointestinal side effects and the current high cost of treatment may limit its use. By contrast, the capsule formulation of itraconazole should be taken with a full meal or with acidic beverages to improve absorption. Acid-blocking agents should be avoided. To ensure adequate drug levels and tissue penetration, serum levels should be obtained after two weeks of therapy [46]. In 2018, a novel formulation of itraconazole, super bioavailable (“SUBA”)-itraconazole was approved by the Food and Drug Administration (FDA) as a 65 mg capsule for the treatment of systemic fungal infections, including pulmonary and extrapulmonary blastomycosis. A recent study showed that SUBA-itraconazole has less variable absorption as compared to conventional itraconazole under fasted conditions. Whether the use of SUBA-itraconazole is associated with superior outcomes in blastomycosis is unknown [108,109].

For patients who do not tolerate itraconazole, alternative options exist; however, these are less effective and/or have limited data to support their use. High-dose fluconazole (400–800 mg daily) and ketoconazole (400–800 mg daily) have been used successfully, though ketoconazole-related side effects are common at high doses, and ketoconazole should generally be avoided [46,110,111]. The roles of voriconazole and posaconazole in the treatment of pulmonary blastomycosis remain poorly understood, with data limited to case reports [53,112].

#### 5.1.2. Moderately Severe to Severe Pulmonary Blastomycosis

For severe disease, the recommended initial treatment is intravenous amphotericin B (lipid formulation of amphotericin B at 3–5 mg/kg per day; or amphotericin B deoxycholate 0.7–1 mg/kg per day) for 1–2 weeks or until clinical improvement, followed by step-down therapy to oral itraconazole (200 mg three times per day for three days, followed by 200 mg twice daily for 6–12 months) [46]. The lipid formulation of amphotericin is preferred due to the lower rates of nephrotoxicity [113]. Patients who require mechanical ventilatory support and develop adult respiratory distress syndrome (ARDS) have a poor prognosis [114]. The use of corticosteroids in this setting remains controversial and unproven, with data limited to case reports [115]. Although not recommended by the IDSA guidelines, adjunctive corticosteroids are recommended on a case-by-case basis for patients with life-threatening pulmonary blastomycosis during the first two weeks of therapy by the American Thoracic Society and some experts [116].

### 5.2. Disseminated/Extrapulmonary Blastomycosis

Extrapulmonary blastomycosis always requires treatment, even if complete tissue resection is anticipated, such as with surgical resection of cutaneous blastomycosis lesions [46]. Identifying whether there is central nervous system (CNS) involvement is a key step to ensure that a treatment regimen is used with adequate CNS penetration. Itraconazole is thought to achieve poor concentrations in the CNS and should generally be avoided [117]. Interestingly, it is used for step-down therapy in CNS histoplasmosis [118]. Similarly, determining the presence of osteoarticular disease is important, as osteoarticular blastomycosis can be more difficult to treat. The IDSA guidelines recommend at least a 12-month treatment duration due to a higher risk of relapse; however, there are little data to support this practice, and most recommendations are primarily based on expert opinion [46,119]. Disseminated disease without CNS involvement is treated the same as pulmonary disease, with severe disease starting with amphotericin upfront and mild–moderate disease focusing on itraconazole alone. High-dose fluconazole (400–800 mg per day) can be used as an alternative option for mild–moderate disease or step-down therapy, but it is less effective [46,110]. The roles of voriconazole and posaconazole are poorly understood.

### 5.3. CNS Blastomycosis

The recommended treatment of CNS blastomycosis is a lipid formulation of amphotericin B (higher doses are more commonly used but with incomplete evidence to support this practice) for 4–6 weeks followed by an azole for at least 12 months and until CNS abnormalities have resolved [46]. Liposomal amphotericin B has been shown in animal models to have superior CNS penetration as compared to alternative amphotericin B formulations, although whether it is the same in humans is not clear [46,120]. The optimal choice of azole following the completion of amphotericin B is poorly understood, with clinical data primarily limited to case reports [46]. The most experience appears to be with the off-label use of voriconazole, which has known intrinsic activity against *Blastomyces* and achieves adequate CSF concentrations [117]. Itraconazole has poor CNS concentration and, thus, is less effective at treating disease at this site, but in some settings, it is commonly used. By contrast, fluconazole has excellent CNS penetration but has less intrinsic activity against blastomycosis [107]. Surgical debridement may be needed in some cases, though more research is needed to define the optimal role and timing of debridement [121].

## 6. Immunosuppressed Patients with Blastomycosis

Immunocompromised patients (patients with HIV/AIDS, recipients of solid organ transplants, and patients with hematologic malignancies) appear to be at greater risk of developing severe disease and respiratory failure, and of dying from blastomycosis [122,123,124]. Intravenous liposomal amphotericin B is used in the same way as described above and is similarly followed by itraconazole. Life-long suppression with itraconazole 200 mg per day should be considered in patients for whom immunosuppression cannot be withheld and/or in patients experiencing relapse despite recommended treatment [46]. Although induction therapy with itraconazole is not recommended, a recent retrospective analysis of patients with proven blastomycosis identified multiple patients with solid organ transplant and malignancies on chemotherapy who were treated successfully with itraconazole monotherapy for the full duration of treatment [124]. Though promising, clinical trial data are needed to determine whether itraconazole, or other azoles, have a role in the initial treatment of blastomycosis in immunocompromised hosts. Furthermore, the close monitoring of drug (itraconazole and immune suppression medications) levels is particularly important in patients who have undergone solid organ transplant.

## 7. Blastomycosis in Pregnancy and Newborns

Intravenous liposomal amphotericin is the mainstay of therapy in blastomycosis affecting pregnant patients and should be continued until after the delivery or resolution of the infection, whichever occurs first. This is based on the possible teratogenic effects of azoles seen in animal studies [46]. There are limited data suggesting that fetal risk is higher than maternal risk in blastomycosis of pregnancy, owing to the potential for trans-placental transmission of *Blastomyces* [125]. Thus, if a mother is confirmed to have blastomycosis during pregnancy, it is recommended that the placenta be examined after delivery for signs of *Blastomyces* infection and that the newborn be monitored for emerging signs and symptoms of blastomycosis. Amphotericin deoxycholate is recommended to treat the newborn if they do become infected [46].

## 8. Alternative Azoles

There are emerging data supporting isavuconazole as an option for blastomycosis in immunocompromised patients, including those with CNS blastomycosis. This drug is appealing due to it having a smaller drug–drug interaction profile than other azoles. A recent retrospective case series of 14 patients with blastomycosis (half of whom were immunocompromised, and half of whom had CNS involvement) treated with isavuconazole demonstrated a 79% cure rate, suggesting that this drug could potentially be used in a broad profile of patients, particularly when other azoles would cause safety or drug interaction issues [126]. Similarly, a retrospective case series of blastomycosis in recipients of solid organ transplants at a single medical center in Wisconsin included a subset of patients treated with voriconazole, posaconazole, or fluconazole. Though not powered to examine differences in outcomes between these therapies, it offers a glimpse into the real-world use of these azoles in patients with blastomycosis, and it suggests that, in the right context, they may be useful alternatives to itraconazole [127]. That being said, there is still a significant need for large-scale clinical trials to strengthen this data.

## 9. Future Research

Significant knowledge gaps exist in our understanding of the evolving epidemiology of blastomycosis, as well as the effectiveness and appropriateness of newer azole medications for this infection. In the United States, blastomycosis is not a reportable disease outside of five states within the Mississippi River valley region [27]. Due to this, epidemiologic data on this infection outside of those states are piecemeal and often limited to case reports. Those epidemiologic studies that have been performed are typically focused on patients presenting to large academic medical centers, potentially skewing the geolocation data to the site of diagnosis rather than the site of exposure [28]. A large, multisite study that decouples the diagnosis and exposure sites for those with symptomatic blastomycosis would provide significant insight into the growing geographic reach of this endemic mycosis. Newer diagnostic methods, notably next-generation sequencing and targeted fungal PCR assay approaches similar to 13 s, are also largely unexplored in blastomycosis (and endemic mycoses in general); these technologies have the potential to provide a low-cost, rapid diagnosis with high specificity and sensitivity, but, unfortunately, they have not had much funding or attention to date.

Though itraconazole is the foundation of treatment for blastomycosis, there are occasionally situations where it is not a viable therapeutic option (intolerance, drug–drug interactions, unavailability, cost, etc.). Experience with other azoles as primary therapy, or as a transition from amphotericin in severe or CNS blastomycosis, is data-poor and mostly based on case reports or studies involving only a very small number of patients, leaving clinicians to make difficult therapeutic decisions without much of a data-driven foothold. Additionally, even when itraconazole is appropriate and available, there are significant questions that remain regarding the ideal length of therapy and the role of adjunctive steroids. Future studies, either focused broadly on the outcomes of patients with endemic mycoses treated with a variety of azole-based regimens or, more narrowly, on a specific azole, would help establish the non-inferiority of these alternative agents and could provide more therapeutic options for clinicians treating difficult blastomycosis cases.

## 10. Conclusions

*Blastomyces* is a significant fungal pathogen endemic to a large swath of the United States, Africa, and potentially India and the Middle East. Though primarily a pulmonary pathogen, *Blastomyces* is capable of causing severe disease throughout much of the body and is of particular concern in the immunocompromised population. As summarized above, diagnosis requires keen attention to the patient’s personal exposure risk epidemiologic factors, and their clinical presentation, as well as microbiologic and other laboratory data, as available. Itraconazole is often the foundation of treatment for mild-to-moderate blastomycosis, with more severe or CNS disease requiring an initial amphotericin phase. Experience with alternative azoles is limited, with voriconazole perhaps having the best evidence. However, other azoles may still be viable options in cases where itraconazole is contraindicated or unavailable. Despite increasing incidence, there remain significant knowledge gaps in the epidemiology and management of this infection—areas where future research needs to be carried out.

## Figures and Tables

**Table 1:** Management of blastomycosis.

Type of Infection	Drug(s) of Choice	Duration of Therapy
Pulmonary, mild to moderate	Itraconazole ^a^	6–12 months
Pulmonary, severe	Induction: Liposomal amphotericin (or amphotericin B deoxycholate) ^b^ Step-down: Itraconazole ^a^	Induction therapy × 1–2 weeks (or until clinical improvement), then oral therapy × 6–12 months.
Disseminated, mild–moderate, no CNS involvement	Itraconazole ^a^	At least 12 months
Disseminated, severe or with CNS involvement	Induction: Liposomal amphotericin (or amphotericin B deoxycholate) ^b^ Step-down: Itraconazole ^a^	Induction therapy × 1–2 weeks (or until clinical improvement), then oral therapy × 6–12 months.
Immunosuppressed, any form of blastomycosis	Induction: Liposomal amphotericin (or amphotericin B deoxycholate) ^b^ Step-down: Itraconazole ^a^	Induction therapy × 1–2 weeks (or until clinical improvement), then oral therapy × 6–12 months (can be continued as lifelong suppression if ongoing immunosuppression) ^c^

CNS: central nervous system. Treatment recommendations based on 2008 IDSA guidelines [46]. ^a^ 200 mg three times daily × 3 days, followed by 200 mg twice daily for remainder of therapy. ^b^ Liposomal formulation dosed at 3–5 mg/kg/day; deoxycholate dosed at 0.7–1 mg/kg/day. ^c^ Lifelong suppression dosed at 200 mg daily.

## Data Availability

Not applicable.
